# Type 1 Interferons and NK Cells Limit Murine Cytomegalovirus Escape from the Lymph Node Subcapsular Sinus

**DOI:** 10.1371/journal.ppat.1006069

**Published:** 2016-12-07

**Authors:** Helen E. Farrell, Kimberley Bruce, Clara Lawler, Rhonda D. Cardin, Nicholas J. Davis-Poynter, Philip G. Stevenson

**Affiliations:** 1 School of Chemistry and Molecular Biosciences and Child Health Research Centre, University of Queensland, Brisbane, Australia; 2 Pathobiological Sciences, School of Veterinary Medicine, Louisiana State University, Baton Rouge, Louisiana, United States of America; 3 Child Health Research Centre, University of Queensland, Brisbane, Australia; University of California Berkeley, UNITED STATES

## Abstract

Cytomegaloviruses (CMVs) establish chronic, systemic infections. Peripheral infection spreads via lymph nodes, which are also a focus of host defence. Thus, this is a point at which systemic infection spread might be restricted. Subcapsular sinus macrophages (SSM) captured murine CMV (MCMV) from the afferent lymph and poorly supported its replication. Blocking the type I interferon (IFN-I) receptor (IFNAR) increased MCMV infection of SSM and of the fibroblastic reticular cells (FRC) lining the subcapsular sinus, and accelerated viral spread to the spleen. Little splenic virus derived from SSM, arguing that they mainly induce an anti-viral state in the otherwise susceptible FRC. NK cells also limited infection, killing infected FRC and causing tissue damage. They acted independently of IFN-I, as IFNAR blockade increased NK cell recruitment, and NK cell depletion increased infection in IFNAR-blocked mice. Thus SSM restricted MCMV infection primarily though IFN-I, with NK cells providing a second line of defence. The capacity of innate immunity to restrict MCMV escape from the subcapsular sinus suggested that enhancing its recruitment might improve infection control.

## Author Summary

Cytomegaloviruses (CMVs) infect most people and are a common cause of fetal damage. We lack an effective vaccine. Our knowledge of human CMV is largely limited to chronic infection, which is hard to treat. Vaccination must target early infection. Related animal viruses therefore provide a vital source of information. Lymph nodes are a bottleneck in murine CMV spread from local to systemic infection. We show that viral passage through lymph nodes is restricted by interferons and NK cells. These defences alone cannot contain infection, but boosting their recruitment by vaccination has the potential to keep infection locally contained.

## Introduction

Human CMV is a ubiquitous pathogen that causes birth defects and harms immunocompromised hosts [1]. Although adaptive immunity normally prevents disease, adaptive immune priming has not prevented infection establishment [2], suggesting that this presents a qualitatively distinct challenge, requiring possibly different immune effectors. Analysing early human infection is made difficult by CMV transmission being sporadic and largely asymptomatic. However CMV infections long pre-date human speciation [3], so different host / virus pairs are likely to share common themes and analogous animal infections can yield key insights. MCMV has particular value for understanding how CMVs work *in vivo*, as its host provides the standard model of mammalian cell biology. MCMV exploits myeloid cells to spread [4, 5], and live imaging shows peripheral to systemic spread via lymph nodes (LN) [6], so LN myeloid cells are likely to be a key target for limiting systemic infection.

Host immunity and viral evasion both influence CMV infection outcomes. Which dominates a given context can be hard to predict. Although MCMV has evolved to infect myeloid cells, myeloid phenotypes are diverse [7] and not all support its spread. SSM police the recycling of extracellular fluid from peripheral tissues back to the blood, capturing viruses from the lymph when it enters the LN subcapsular sinus [8, 9]. Viral replication in SSM potentially delivers an amplified load. Therefore this must be curtailed, or at least slowed sufficiently for other defences to come into play. In addition SSM must protect the FRC that line the subcapsular sinus, and are targeted for example by Ebola virus [10]. While pathogen absorption by SSM can protect down-stream targets such as the blood, it is unlikely to prevent FRC infection as they present a large surface area in the same site [11]. Thus the SSM barrier must be active as well as adsorptive.-In this context SSM would capture viruses to sample rather than cleanse the lymph, and would communicate danger signals, for example to NK cells [12, 13]. The subcapsular sinus is a prominent site of type I interferon (IFN-I) transcription [14] and IFN-I stimulates NK cells [15], so it could contribute. SSM also communicate directly with B cells [16]. T cell-dependent antibody responses take several days to become effective, but T cell-independent antibody responses might protect SSM and FRC in some settings.

The anti-viral functions of SSM have been explored by injecting mice with xenogenic pathogens such as Vesicular Stomatitis Virus (VSV) [17]. Footpad-inoculated (i.f.) VSV induces IFN-I, which protects neurons against pathological infection [18]. Whether SSM respond strongly to IFN-I is unknown. VSV productively infects both SSM and splenic marginal zone macrophages (MZM), which capture viruses from the blood. Productive MZM infection is associated with a muted response to IFN-I [19]. This protects by inducing neutralizing antibodies. However VSV is unusually susceptible to neutralization by T cell-independent antibodies [20]. Viral evasion is often compromised in xenogenic infections, and the best known human rhabdovirus infection—rabies—spreads predominantly via neurons, so how far VSV in mice constitutes a general case is unclear.

MCMV has evolved to infect mice and is not susceptible to T cell-independent antibody responses [21]. Nonetheless SSM restrict its spread [6]. They also restrict Murid Herpesvirus-4 (MuHV-4), an evasive gamma-herpesvirus [22]. How is unknown. IFN-I may contribute as it has anti-MCMV [23] and anti-MuHV-4 activity [24], despite viral evasion. After intraperitoneal (i.p.) MCMV inoculation, IFN-I signals to NK cells and DC [25]. I.p. MCMV prominently infects the liver, and IFN-I suppresses viral lytic gene expression in *in vitro* propagated liver cells [26]. However the failure of hepatocytes to spread infection *in vivo* [27] makes unclear the relevance of liver infection to normal pathogenesis. Herpesviruses normally enter at peripheral sites, whereas i.p. virions reach the blood directly [28], bypassing SSM. We show that SSM are a key site of IFN-I-mediated defence against MCMV. When IFN-I signalling was blocked, lymph-borne MCMV spread rapidly to systemic sites. NK cells provided a second line of defence but at the cost of tissue damage. Thus, an SSM-centered IFN-I response was crucial to limit MCMV dissemination.

## Results

### IFNAR blockade increases MCMV spread in BALB/c mice

**Fig 1:**
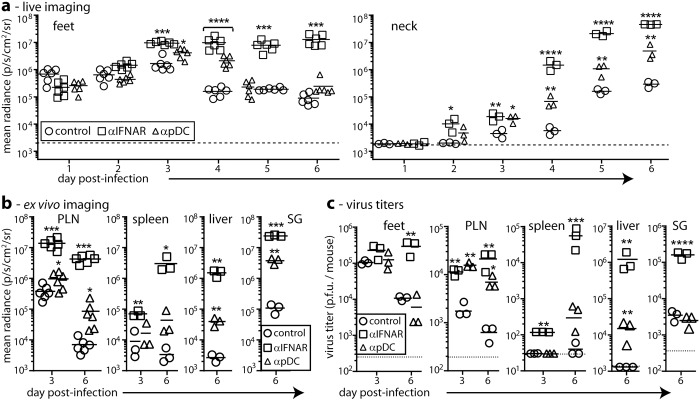
IFNAR blockade increases MCMV dissemination from a peripheral site. **(a).** BALB/c mice were given IFNAR blocking (αIFNAR) or pDC depleting (αpDC) antibodies in PBS, or PBS only (control), then given MCMV-LUC i.f. (10^6^ p.f.u.). We tracked infection by luciferin injection and live imaging of light emission (radiance = photons/sec/cm^2^ /steradian). Bars show means, other symbols show individuals. Both αIFNAR and αpDC significantly increased luciferase signals in the feet (footpad + PLN) and in the neck (salivary gland) from day 3, with αIFNAR having a significantly greater effect. After day 4, αpDC only affected neck signals. (Student’s two-tailed unpaired t-test; *p<0.05, **p< 0.01, ***p<0.001, ****p<0.0001). The dotted lines show assay sensitivity limits. **(b).** Mice were treated and infected as in (**a)**, and organs harvested 3 or 6 days later for *ex vivo* luciferase imaging. Liver and salivary gland signals were not detected at day 3. The Y axis baselines correspond to assay sensitivity limits. Significant signals above the controls are indicated according to the scheme in (**a)**. (**c).** The organs from (**b)** were plaque assayed for infectious virus. Bars show means, other symbols show individual organs. Dotted lines show assay sensitivity limits where above the Y axis baseline. Titers significantly above those of controls are indicated. Significant signals above the controls are indicated according to the scheme in (**a)**.

We hypothesized that IFN-I contributes to SSM restricting MCMV infection. We first tracked by live imaging how IFNAR blockade affects MCMV spread. We gave BALB/c mice IFNAR blocking antibody or not i.p. then MCMV-LUC i.f. and imaged them daily for luciferase expression (Fig 1a).

Live image signals from untreated infected mice were evident in the feet from day 1, and in the neck days 4–5. IFNAR blockade significantly increased foot signals from day 3 and neck signals from day 4. Plasmacytoid DC (pDC) produce IFN-I [29], and prior pDC depletion with a bst-2-specific antibody also increased live image signals, but it had less effect than IFNAR blockade. This was consistent with genetic pDC depletion having only a modest effect on MCMV spread after i.p. inoculation [30].

Live image signals are comparable between mice for the same organs, but less so between different organs because overlying tissues cause site-dependent signal attenuation. Signals from adjacent organs can also be hard to distinguish. Therefore to understand better how IFNAR blockade affected MCMV passage through LN, we dissected mice 3 and 6 days after i.f. MCMV-LUC and imaged organs *ex vivo* (Fig 1b). IFNAR blockade increased signals in multiple organs at day 6, and in popliteal LN (PLN) and spleens at day 3. Depleting pDC also increased luciferase signals, predominantly at day 6, but had less effect than IFNAR blockade.

To correlate luciferase expression with virion production, we measured virus titers in the same organs (Fig 1c). They showed similar trends: IFNAR blockade increased titers in many organs at day 6 and in just PLN and spleens at day 3. pDC depletion generally had less effect, increasing titers in PLN but not in the feet or salivary glands and only modestly in the liver and spleen. Thus Consistently the PLN appeared to be an important site of IFN-I-mediated anti-MCMV defence.

### IFNAR blockade acts early in infection

**Fig 2:**
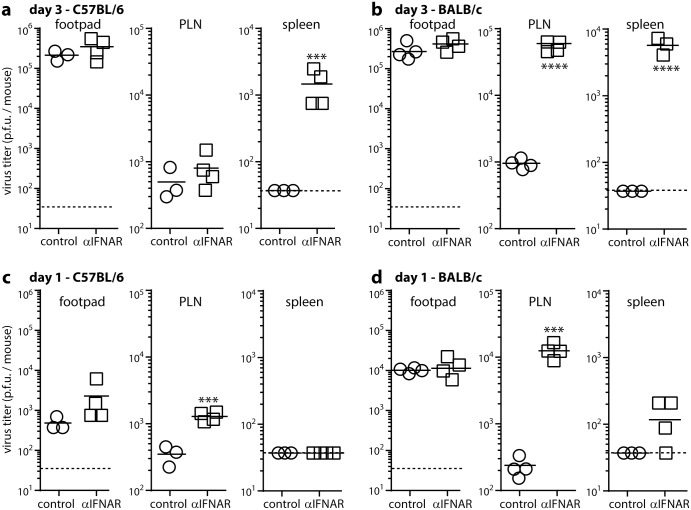
IFNAR blockade increases early LN infections of C57BL/6 and BALB/c mice. C57BL/6 (**a**, **c**) and BALB/c (**b**, **d**) mice were given IFNAR blocking antibody (αIFNAR) or not (control) i.p., then MCMV i.f. (10^6^ PFU). Infectious virus was plaque assayed 3 days (**a**, **b**) or 1 day later (**c**, **d**). Bars show means, other symbols show individual mice. Dotted lines show assay sensitivity limits. Significant differences were calculated and are indicated according to the scheme of Fig 1.

We compared next how IFNAR blockade affected MCMV spread in C57BL/6 mice as an independent strain. BALB/c and C57BL/6 mice were each given anti-IFNAR blocking antibody or not then i.f. MCMV. The C57BL/6 NK receptor Ly49H recognizes directly the MCMV m157 [31–33]. m157 is polymorphic among MCMV strains; many variants do not bind Ly49H [34]; and m157^+^ virus passage in C57BL/6 mice selects m157^-^ mutants [35]; so m157^-^ MCMV is probably more representative of the viruses that colonize Ly49H^+^ mice, and in these experiments we used MCMV in which a GFP cassette interrupts m157 (MCMV-GR; Fig 2).

After 3 days IFNAR blockade increased viral titers in the spleens of both mouse strains (Fig 2a and 2b). It significantly increased day 3 PLN titers in BALB/c but not C57BL/6 mice (Fig 2a and 2b). I.f. MCMV reaches the spleen via the PLN [6], so we also looked earlier for increased PLN infection, and at day 1 observed this in both strains (Fig 2c and 2d). At day 1 spleen infection was low or undetectable and footpad infection did not differ significantly from controls. Therefore IFNAR blockade increased PLN infection independently of footpad infection and before spleen infection, with C57BL/6 mice showing a smaller, less sustained increase than BALB/c mice.

### IFNAR blockade increases SSM and FRC infections

**Fig 3:**
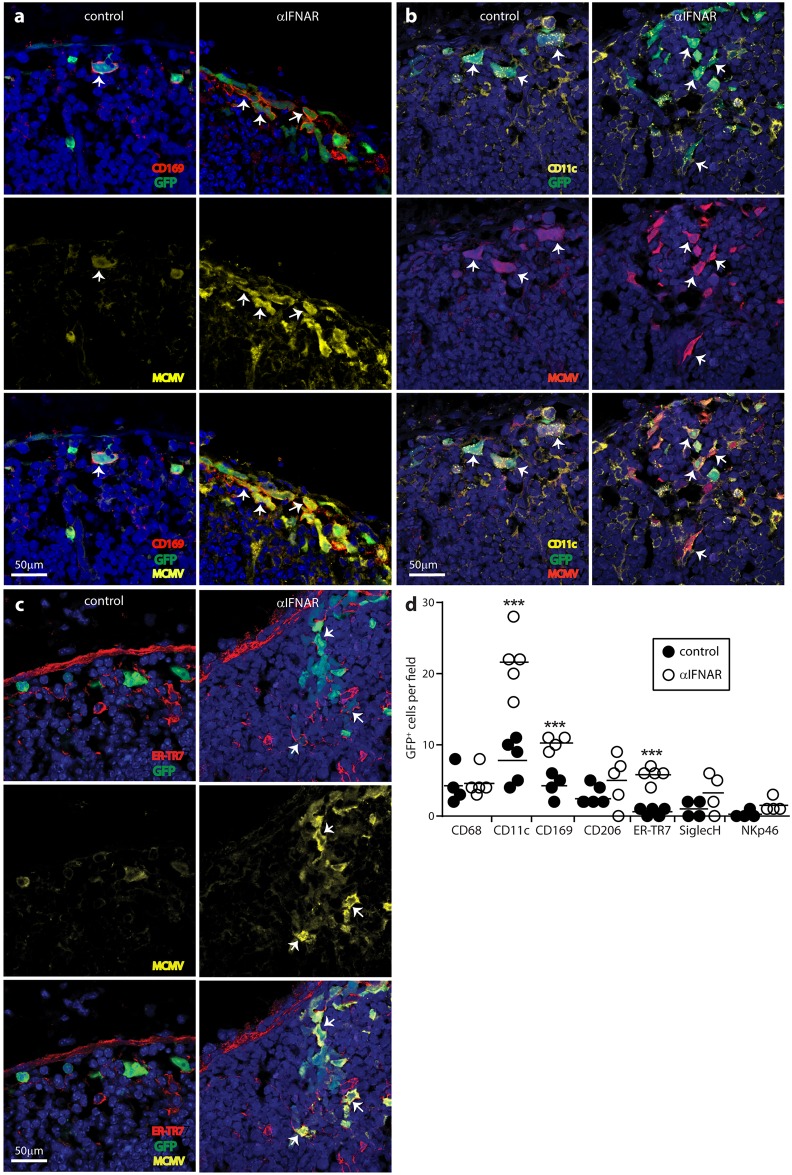
IFNAR blockade increases SSM and FRC infections. C57BL/6 mice were given IFNAR blocking antibody (αIFNAR) or not (control), then MCMV-GR i.f. (10^6^ p.f.u.). PLN taken 1 day later were stained for viral GFP (GFP) and lytic antigens (MCMV), plus the SSM marker CD169 (**a**), the SSM/DC marker CD11c (**b**) or the FRC marker ER-TR7 (**c**). Nuclei were stained with Hoechst 33342 (blue). Arrows show example infected SSM and FRC. Both were more numerous after αIFNAR. In (**d**) we counted cells across 4–5 fields of view for PLN sections from each of 3 mice per group. Circles show individual means, bars show group means. Significant differences are indicated (Student’s two tailed unpaired t-test; ***, P<0.001; ****, P<0.0001). We quantified infection also for CD68^+^ (pan-macrophage/DC), CD206^+^ (mannose receptor, absent from SSM), SiglecH^+^ (pDC) and NKp46^+^ (NK) cells.

MCMV infects multiple cell types. To identify the source of increased virus titers in the PLN following IFNAR blockade, we gave mice anti-IFNAR or not, infected them with MCMV-GR and 1 day later stained PLN sections for viral GFP and lytic antigens (Fig 3).

To focus on common, conserved events rather than increases that might be mouse strain-specific, we analysed the less extensive infection of C57BL/6 mice. IFNAR blockade increased viral GFP and lytic antigen expression around the subcapsular sinus (Fig 3a, 3b and 3c). Most viral GFP^+^ cells (approximately 75%) were viral lytic antigen^+^ and most lytic antigen^+^ cells were GFP^+^, suggesting that most infection in the PLN was lytic. Higher CD169^+^GFP^+^ cell counts indicated that IFN blockade increased SSM infection (Fig 3a and 3d). Higher CD11c^+^GFP^+^ cell counts (Fig 3b and 3d) were consistent with more SSM infection as SSM express CD11c. More DC infection was also possible. Higher ER-TR7^+^GFP^+^ cell counts (Fig 3c and 3d) indicated that IFNAR blockade also increased FRC infection. Taken together, IFNAR blockade did not increase early footpad infection (Fig 2) but increased lytic infection in the SSM and FRC of PLN.

**Fig 4:**
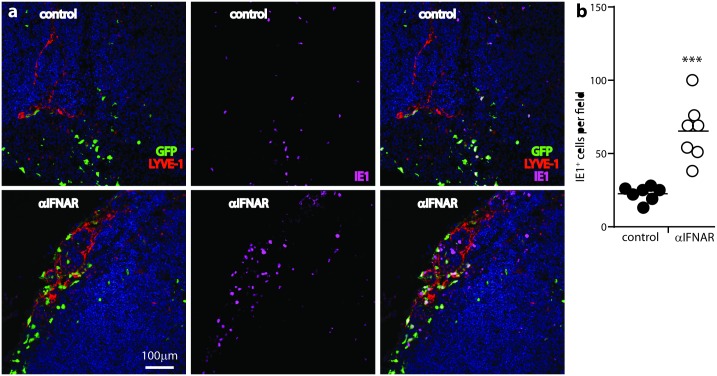
IFNAR blockade increases MCMV lytic infection in LN. C57BL/6 mice were given IFNAR blocking antibody (αIFNAR) or not (control), then MCMV-GR i.f. (10^6^ p.f.u.). PLN taken 1 day later were stained for viral GFP, MCMV IE1 antigen, and LYVE-1 (lymphatic endothelium). (**a)** shows examples of staining. (**b)** shows quantitation, counting IE^+^ cells for 7 fields of view in PLN sections from each of 3 mice per group. Circles show individual means, bars show group means. αIFNAR significantly increased IE^+^ cell numbers (Student’s two tailed unpaired t-test with Welch’s Correction; ***, P<0.001).

As a further measure of lytic infection, we stained PLN sections for the MCMV IE1 antigen (Fig 4a).

IE1 is not abundant in virions and is expressed in the nuclei of lytically infected cells, so its expression clearly distinguishes lytic infection from antigen uptake. IFNAR blockade significantly increased IE1^+^ cell numbers in the PLN (Fig 4b). Thus, lytic antigen, IE1 and viral GFP expression all showed IFNAR blockade increasing MCMV infection of the LN subcapsular sinus.

### NK cells constitute a second, IFN-I-independent line of anti-MCMV defence

**Fig 5:**
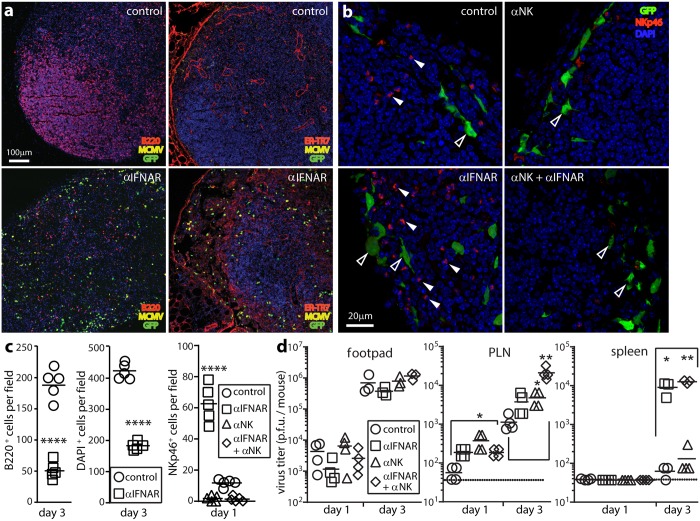
IFN-I-independent NK cell recruitment aids infection control. **(a).** C57BL/6 mice were given IFNAR blocking antibody (αIFNAR) or not (control) then i.f. MCMV-GR (10^6^ p.f.u.). 3 days later PLN were stained for viral GFP, lytic antigens (MCMV), and B cells (B220) or FRC (ER-TR7). Nuclei were stained with DAPI. αIFNAR reduced B220 and DAPI staining, and disorganized ER-TR7 staining, implying loss of LN cellularity and structure. **(b).** C57BL/6 mice were given αIFNAR, depleted of NK1.1^+^ cells with mAb PK136 (αNK), given both treatments, or given neither (control). All were then given i.f. MCMV-GR (10^6^ p.f.u.). 1 day later PLN were stained for viral GFP and NKp46^+^ NK cells. Nuclei were stained with DAPI. Closed arrows show example NKp46^+^ cells. Open arrows show example GFP^+^ infected cells. **(c).** Quantitation of staining as illustrated in (**a)** and (**b)**, across 7 fields of view for sections from each of 5 mice per group, showing significant loss of LN cellularity in IFNAR treated-mice (B220^+^, DAPI^+^) and significant NK cell recruitment (NKp46^+^) after αIFNAR and effective NK cell depletion by mAb PK136. Bars show group means, other symbols show mean counts of individual mice. (****, P<0.0001; Student’s two-tailed unpaired t-test). **(d).** Organs of mice treated as in (**b)** were plaque assayed for infectious virus. Bars show means, other symbols show individual mice. Significant differences are indicated (Student’s two tailed unpaired t-test; *p<0.05, **p<0.01). Dotted lines show assay sensitivity limits.

To understand why IFNAR blockade did not continue to increase PLN virus titers in C57BL/6 mice after day 1, we examined day 3 PLN sections (Fig 5).

IFNAR blockade increased viral GFP and lytic antigen expression, as at day 1. However there was also widespread tissue destruction, with less B cell (B220) and DAPI staining, and disruption of the ER-TR7^+^ LN architecture (Fig 5a). The relatively low PLN titers at day 3 suggested that these changes were immunopathological rather than a result of -viral cytopathology.

NK cells were a possible mediator, and day 1 PLN sections of IFNAR-blocked mice showed a significant increase in NKp46^+^ cell numbers that was abolished by NK1.1^+^ cell depletion (Fig 5b and 5c). The increase in numbers seemed likely to reflect NK cell recruitment, as 1 day allowed little time for local proliferation. Although IFN-I can recruit NK cells [36], NKp46^+^ cell recruitment into MCMV-infected PLN increased when IFN-I signalling was blocked, reflecting presumably a dominant inflammatory effect of higher viral loads (Fig 5b and 5d).

**Fig 6:**
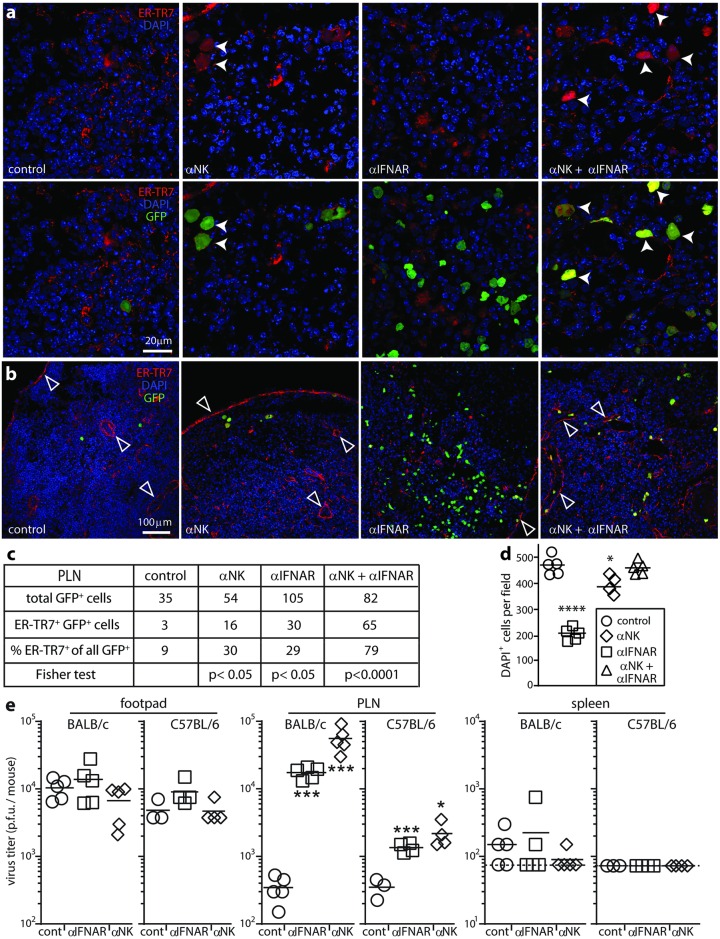
NK cells eliminate ER-TR7^+^ infected cells and cause tissue damage. **(a).** C57BL/6 mice were given IFNAR blocking antibody (αIFN), depleted of NK1.1^+^ cells (αNK), given both treatments, or given neither (control). All were then given i.f. MCMV-GR (10^6^ p.f.u.). 1 day later PLN sections were stained for viral GFP and the FRC marker ER-TR7. Nuclei were stained with DAPI. Arrows show example infected ER-TR7^+^ cells, which were abundant after αNK. **(b).** Low magnification PLN images of mice treated as in **(a),** but at 3 days post-infection, showing reduced DAPI staining and altered ER-TR7 staining (arrows) after αIFN, and restoration of these changes by αNK. **(c).** Counts of infected cells as shown in **a**, summing across 6 fields of view from sections of 3 mice per group. Statistical comparison by Fisher's Exact Test is between each treatment group and the control. **(d).** counts of DAPI^+^ nuclei as shown in **b**, for 5 fields of view from sections of 3 mice per group. Bars show means, other symbols show individual fields of view. IFNAR blockade significantly reduced the density of DAPI^+^ nuclei compared to control PLN but not when NK cells were also depleted. NK depletion alone had a small effect (*p<0.05, ***p<0.001; Student’s two tailed unpaired t test). (**e).** BALB/c and C57BL/6 mice were given αIFN, depleted of asialoGM1^+^ cells (αNK), or left untreated (cont). All were then given i.f. MCMV-GR (10^6^ p.f.u.). Organs were plaque assayed for infectious virus 1 day later. Bars show means, other symbols show individuals. Dashed lines or axis baselines show assay sensitivity limits. Significant differences relative to the controls for each mouse strain are indicated (*p<0.05, ***p<0.001; Student’s two tailed unpaired t-test).

NK cell depletion increased virus titers in C57BL/6 PLN at days 1 and 3, in both IFNAR-blocked and control mice, without significantly increasing titers in footpads or spleens (Fig 5d). As with IFNAR blockade, day 1 PLN sections of NK-depleted mice showed more infected ER-TR7^+^ cells (Fig 6a and 6c).

Therefore both IFN-I and NK cells restricted FRC infection. NK cell depletion increased PLN virus titers as much as IFNAR blockade (Fig 5d) but had less effect on viral GFP^+^ cell numbers (Fig 6a and 6b). Not all GFP^+^ cells were lytic antigen^+^ (Fig 4), so GFP^+^ /lytic antigen^-^ cells may be poor NK cell targets. NK cell depletion with anti-asialo-GM1 antibody significantly increased day 1 PLN virus titers in both BALB/c and C57BL/6 mice, with a greater effect in BALB/c (Fig 6e), so BALB/c NK cells also helped to control early infection.

**Fig 7:**
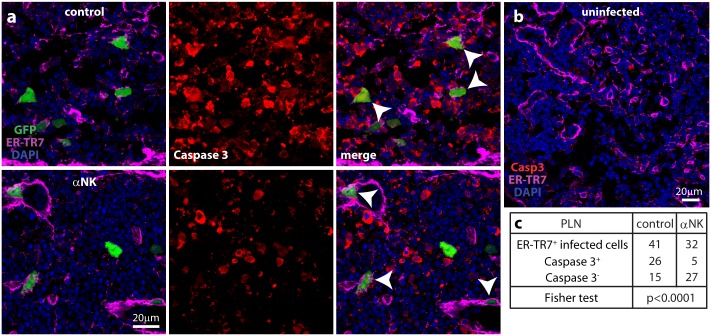
Caspase 3 expression in MCMV-infected PLN. **(a).** C57BL/6 mice were depleted of NK1.1^+^ cells (αNK) or not (control), then given i.f. MCMV-GR (10^6^ p.f.u.). 1 day later PLN sections were stained for viral GFP, the FRC marker ER-TR7 and the apoptotic cell marker caspase 3. Nuclei were stained with DAPI. Arrows show example FRC, caspase 3^+^ in the control and caspase 3^-^ in the αNK. (**b).** Staining of an uninfected PLN showed negligible caspase 3 expression. **(c).** Counting samples of mice treated as in **a**, across 12 sections from 4 PLN, showed by Fisher's Exact Test a significant reduction in FRC caspase 3 expression after αNK.

There was no loss of DAPI staining in dual NK-depleted/IFNAR-blocked PLN of C57BL/6 mice at day 3 (Fig 6d), despite higher virus titers (Fig 5d). This result suggested that NK cells caused the PLN damage. Infected PLN of IFNAR-blocked mice showed extensive expression of the apoptotic cell marker caspase 3 (Fig 7a), which was negligible in uninfected PLN (Fig 7b). Most viral GFP^+^ FRC were caspase 3^+^; when NK cells were depleted, most were caspase 3^-^ (Fig 7a and 7c). Therefore NK cell recruitment into the PLN led to apoptosis in infected FRC.

### Comparing IFNAR blockade with SSM depletion

**Fig 8:**
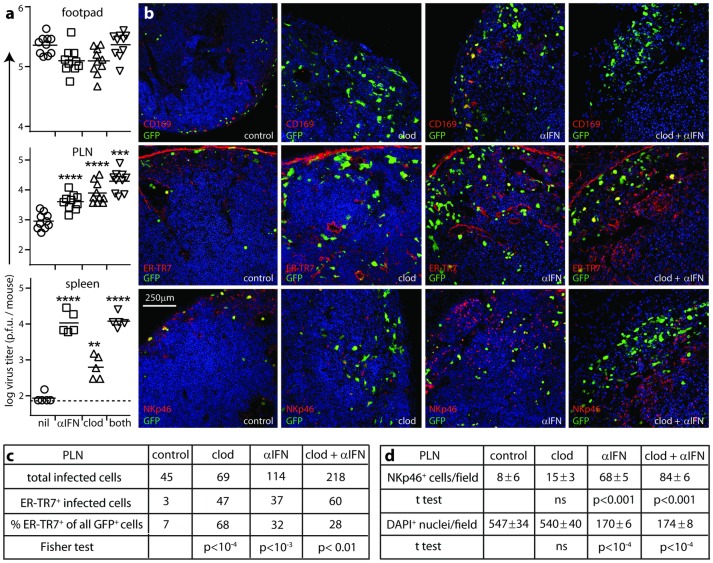
Comparison of SSM depletion and IFNAR blockade effects on PLN infection. **(a).** C57BL/6 mice were depleted of SSM with liposomal clodronate (clod), given IFNAR blocking antibody (αIFN), given both treatments, or neither (control), then infected i.f. with MCMV-GR (10^6^ p.f.u.). 3 days later, organs were plaque-assayed for infectious virus. Horizontal bars show means, other symbols show individuals. Dashed lines show assay sensitivity limits where above the y axis baseline. Significant increases above the control group are indicated (**p<0.01, ***p<0.001, ****p< 0.0001; Student’s two tailed unpaired t-test). In PLN, αIFN and clod treatments did not give significantly different titers (p>0.5), and each alone increased titers significantly less than both together (p<0.05). (**b).** Mice were treated and infected as in (**a)**. Day 3 PLN sections were stained for viral GFP and markers of SSM (CD169), FRC (ER-TR7) or NK cells (NKp46). Nuclei were stained with DAPI. All groups showed loss of CD169 expression. Clod treatment increased FRC infection but caused little of the NK cell infiltration, loss of DAPI staining and ER-TR7 disruption seen with IFNAR blockade. (**c).** Counts of infected cells as shown in (**b)**, summing across 5 fields of view from sections of 4 mice per group. Statistical comparison is between each treatment group and the control using Fisher's Exact Test. (**d).** Counts of NKp46^+^ and DAPI^+^ cells as shown in (**b)**, comparing mean counts per field of view for 6 fields of view from sections of 3 mice per group. Statistical comparisons were with the control group. ns = not significant (p>0.05).

Like IFNAR blockade (Fig 1), SSM depletion accelerates MCMV spread from the PLN to the spleen [6]. Thus we hypothesized that IFN-I production by SSMs restricted PLN infection. To compare SSM depletion with IFNAR blockade, we depleted SSM with i.f. liposomal clodronate, blocked IFNAR, gave both treatments, or gave neither, then infected all the mice i.f. with MCMV (Fig 8).

SSM depletion and IFNAR blockade both increased day 3 PLN and spleen infections without increasing footpad infections (Fig 8a). In PLN they gave similar increases and when combined gave an additive increase. Therefore IFN-I did not come just from SSM—notably pDC depletion also increased virus titers (Fig 1)—and it did not act just on SSM, consistent with its protection of FRC (Fig 5). The additive increase implied also that IFN-I was not the only SSM defence, consistent with a report of innate effector recruitment via IL-18 [37]. Nonetheless the additive effect was not large, so IFN-I signalling appeared to be a major component of SSM-mediated MCMV restriction.

*In situ* analysis of day 3 PLN showed markedly increased FRC infection after SSM depletion by clodronate loaded liposomes (“clod”; Fig 8b and 8c). However this increased FRC infection recruited relatively few NK cells (Fig 8d). Thus, although NK cells controlled FRC infection, their recruitment did not depend on FRC infection. IFN-I signalling was not essential to recruit NK cells either (Fig 8d). In fact it limited NK cell recruitment and preserved PLN cellularity, presumably by reducing virus loads (Fig 8c and 8d). NK cell recruitment was maximal after SSM depletion plus IFNAR blockade. Thus, non-SSM myeloid cells such as medullary sinus and medullary cord macrophages and DC [38], which are not depleted by i.f. liposomal clodronate [22], may also produce the necessary cytokines, and the multiplicity of sufficient signals meant that NK recruitment correlated primarily with total virus load. This recruitment was then associated with a reduction in ER-TR7^+^ cell infection and a general loss of LN cellularity.

While SSM depletion alone increased splenic infection, SSM depletion plus IFNAR blockade gave no increase beyond IFNAR blockade alone (Fig 8a). I.f. liposomal clodronate does not deplete MZM [39], so here it could increase infection only by increasing seeding, which IFNAR blockade did already, with IFN-I limiting MCMV spread in both the PLN and the splenic MZ.

### Tracking MCMV passage through SSM

**Fig 9:**
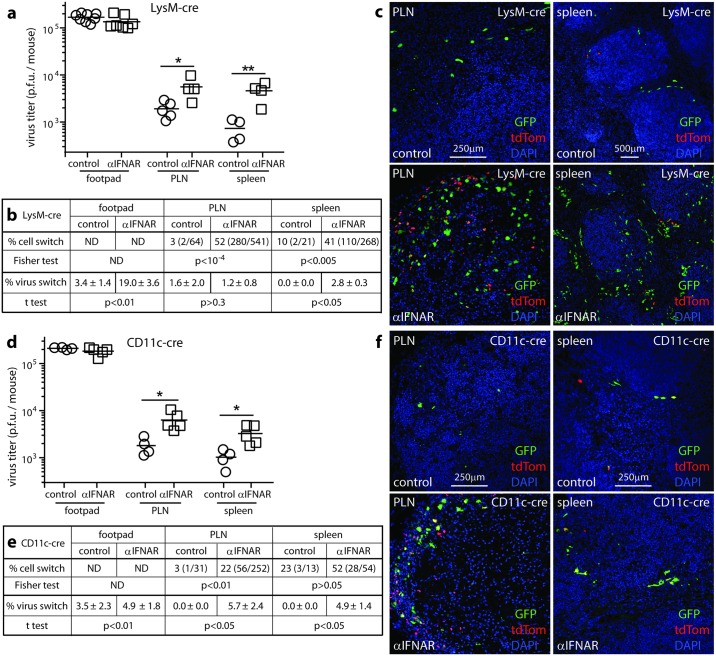
Non-myeloid cells produce most of the infectious virus in IFNAR-blocked mice. **(a,d).** LysM-cre (**a-c**) or CD11c-cre mice (**d-f**) were given IFNAR blocking antibody (αIFNAR) or not (control) then MCMV-GR i.f. (10^6^ p.f.u.). Footpads, PLN and spleens harvested 3 days later were plaque assayed for infectious virus. Squares and circles show individuals, horizontal bars show means. Significant differences are indicated (Student’s two tailed unpaired t-test; *p<0.05, **p<0.01). **(b,e).** Switched (red) and unswitched (green) infected cells were counted on PLN and spleen sections for 5 fields of view per mouse from each of 4 mice per group. The numbers give the % cell switching and in parentheses the switched and total infected cell counts. The switching rates αIFNAR and control mice were compared for each site by Fisher's Exact Test. We also determined below the % virus switching (infectious) recovered from each mouse. The numbers give mean ± SEM for each group. The αIFNAR and control results were compared (Student’s two tailed unpaired t test). **(c,f).** Example viral GFP and tdTomato staining is shown for infected PLN of control and αIFNAR mice. Nuclear DAPI staining shows the loss of cellularity in PLN of αIFNAR mice.

SSM expression of lysM [22] and CD11c [6] allows their MCMV production to be tracked by floxed virus recombination in lysM-cre and CD11c-cre mice. We gave mice anti-IFNAR antibody or not, then i.f. MCMV-GR, which cre switches from GFP to tdTomato expression (Fig 9).

In lysM-cre mice, IFNAR blockade increased MCMV-GR switching in footpads at 3 days (Fig 9b, % virus switch). However the proportion of switched virus in PLN and spleens remained low. Thus, day 3 PLN infection derived from either earlier footpad infection (Fig 2) or inoculated virions [6]. IFNAR blockade did increase PLN virus production by lysM^+^ cells, because total titers increased. However the % switched showed little change, so virus production by lysM^-^ cells also increased (Fig 9a).

In contrast to low switching rates of infectious virus in the PLN and spleen, IFNAR blockade increased the fluorochrome switching of infected cells on tissue sections (% cell switch; Fig 9b and 9c). Infecting CD11c-cre mice gave similar results (Fig 9d–9f), confirming the low virus productivity of SSM and ruling out LysM^-^CD11c^+^ DC as the source of unswitched virus in IFNAR-blocked lysM-cre mice. Whereas floxed MuHV-4 [40] and Herpes simplex virus type 1 [41] show substantial switching in lysM-cre and CD11c-cre mice after IFNAR blockade, unswitched MCMV from cre^-^ cells such as FRC seemed to dilute out switched SSM virus regardless of IFNAR blockade.

To test this idea further, we gave lysM-cre mice IFNAR blocking antibody, optionally also depleted NK cells, (to preserve FRC) then infected the mice i.f. with MCMV-GR. Three days later we recovered virus from footpads, PLN and spleens and quantified fluorochrome switching.

**Fig 10:**
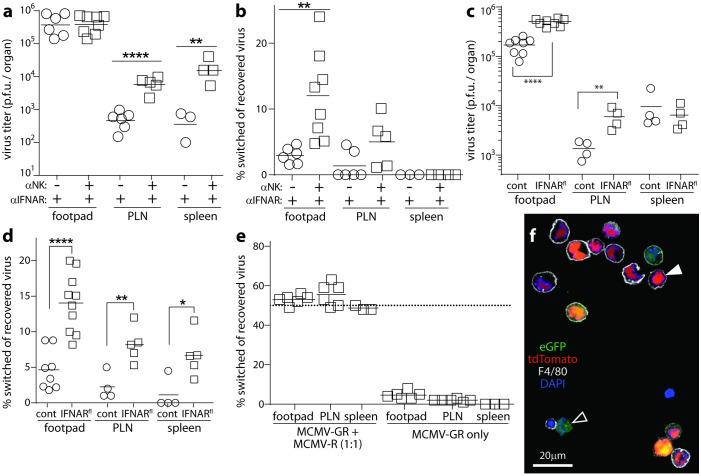
Limited MCMV replication in SSM suggests that they function mainly to limit FRC infection. **(a).** LysM-cre mice were given IFNAR blocking antibody (αIFNAR), without or without additional NK cell depleting antibody (αNK). They were then given MCMV-GR i.f. (10^6^ p.f.u.). Footpads, PLN and spleens harvested 3 days later were plaque assayed for infectious virus. Squares and circles show individuals, horizontal bars show means. Significant differences are indicated (Student’s two tailed unpaired t-test; *p<0.05, **p<0.01, ****p<0.0001). **(b).** Viruses recovered in **(a)** were typed as green or red fluorescent. Squares and circles show the cre-driven green to red switching rates of individual mice, horizontal bars show means. Significant differences are indicated (Student’s two tailed unpaired t-test with Welch’s correction; **, P<0.01) **(c).** LysM^+/cre^ (cont) and LysM^+/cre^IFNAR^flox/flox^ (IFNAR^fl^) mice were depleted of NK cells then infected i.f. with MCMV-GR (10^6^ p.f.u.). Virus titers and comparisons were determined as in **(a)**. **(d).** The viruses recovered in **(c)** were assayed for green to red switching. Significant differences were calculated according to **(b)** and are indicated (*, p<0.05; **, P<0.01; ****, P<0.0001). **(e).** The floxed colour switch virus (MCMV-GR) and its switched derivative (MCMV-R) were mixed 1:1 and given i.f. to lysM-cre mice (10^6^ p.f.u.). As a control, mice were given MCMV-GR only. Viruses recovered after 3 days were assayed for green / red switching. The switching of MCMV-GR was <5%. The mixed viruses remained 1:1 (50% switched). Therefore the low switching of MCMV-GR was not due to poor fitness of the switched virus. **(f).** MCMV-GR was given i.p. to lysM-cre mice (10^6^ p.f.u.). Two days later peritoneal macrophages were recovered by lavage, identified by staining for F4/80, and examined for viral fluorochrome expression. The closed arrow shows an example switched macrophage, with red nuclear fluorescence. The open arrow shows an example unswitched infected cell (F4/80^-^) with cytoplasmic GFP fluorescence. Some F4/80^+^ cells showed residual green fluorescence in addition to red fluorescence, but >90% of fluorescent F4/80^+^ cells were switched.

On a background of IFNAR blockade, additional NK cell depletion significantly increased virus titers in PLN and spleens (Fig 10a), but it increased virus switching only in footpads. In fact, virus switching decreased as infection progressed from PLN to spleens, indicating an increased contribution of non-myeloid cells to virus production at each stage (Fig 10b).

To preserve non-myeloid IFN-I signalling, we infected lysM^+/cre^IFNAR^flox/flox^ mice, which inactivate IFNAR specifically in lysM^+^ cells. These mice, and lysM-cre controls with IFNAR intact, were depleted of NK cells then given MCMV-GR i.f. (Fig 10c and 10d). Virus titers increased in the floxed IFNAR mice, and virus switching, although still low, was significantly more than in the controls. Thus, IFNAR inactivation just in lysM^+^ cells partly offset the diluting effect of non-myeloid (FRC) infection.

The specificity of the block is probably limited by a lack of positive feedback through IFNAR reducing IFN-I production by lysM^+^ SSM in lysM^+/cre^IFNAR^flox/flox^ mice [42], and thereby reducing IFN-I signalling to lysM^-^ FRC. Virus switching was not limited by poor fitness of the switched virus in lysM-cre mice, as i.f. inoculation of a mixture of switched and unswitched viruses gave equal recovery of each from footpads, PLN and spleens (Fig 10e). We observed also equal recovery of mixed switched and unswitched viruses from the liver and spleen after i.p. inoculation, and from the lungs of C57BL/6 mice after i.n. inoculation (data not shown). Nor was the switching insensitive, as i.p. inoculation of lysM-cre mice gave >90% switching in F4/80^+^ peritoneal macrophages (Fig 10f). Rather MCMV appeared inherently to restrain its lytic replication in myeloid cells and replicate readily in FRC.

### IFNAR blockade increases direct infection of ER-TR7^+^ cells

**Fig 11:**
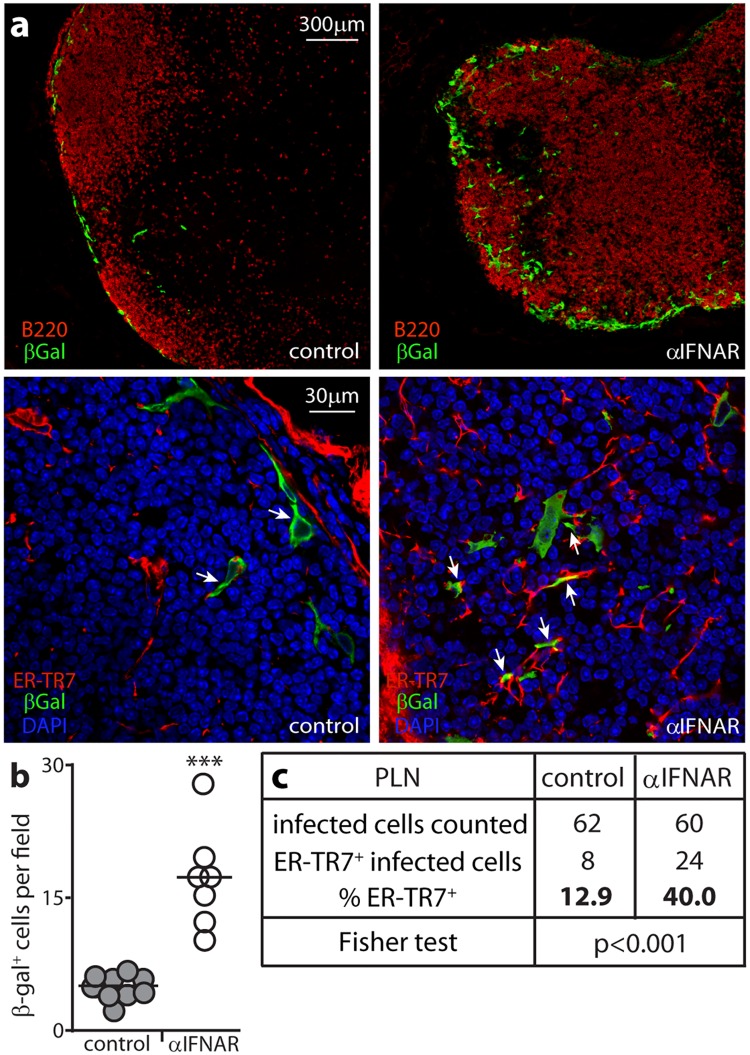
IFN-I protects FRC against direct MCMV infection. **(a).** C57BL/6 mice were given IFNAR blocking antibody (αIFNAR) or not (control) then infected i.f. with MCMV in which a β-galactosidase (βgal) expression cassette disrupts the essential coding sequence of gL (10^6^ p.f.u.). 1 day later PLN sections were stained for βgal to identify infection and for B cells (B220, upper panels) or for FRC (ER-TR7) and with DAPI (lower panels). Arrows in the lower panels show example infected FRC. (**b).** βgal^+^ cells were counted for 6 fields of view from sections of 3 mice per group. Circles show field counts, bars show means. IFNAR blockade significantly increased infection (***p<0.001; Student’s two tailed unpaired t-test with Welch’s Correction). **(c).** The proportion that were ER-TR7^+^ was determined for at least 60 βgal^+^ cells per group (counting more sections from the controls to reach this total). Fisher's Exact Test showed a significantly higher proportion of infected cells was ER-TR7^+^ after IFNAR blockade.

I.f. MCMV directly infects SSM [6]. If it reached FRC via SSM, IFN-I could protect FRC by limiting SSM infection; but if it reached FRC directly, they must be protected directly. To look for direct FRC infection we gave mice IFNAR blocking antibody or not, then gL^-^ MCMV i.f. (Fig 11).

gL is essential for MCMV membrane fusion, so gL^-^ virus is propagated in gL^+^ cells. The pseudotyped gL^+^ virions are infectious but without complementation do not produce infectious progeny. Thus *in vivo*, gL^-^ MCMV infects just once. The gL coding sequence was disrupted by a β-galactosidase (βgal) expression cassette, allowing infected cells to be identified by staining for βgal. Both control and IFNAR-blocked mice had βgal^+^ cells around the subcapsular sinus (Fig 11a). The latter had significantly more per field of view (Fig 11b). Most βgal^+^ cells were SSM. However both control and IFNAR-blocked mice also had βgal^+^ER-TR7^+^ FRC. A significantly higher proportion of βgal^+^ cells were ER-TR7^+^ after IFNAR blockade (Fig 11c). Therefore i.f. MCMV directly infected both SSM and FRC; and IFN-I suppressed FRC infection more than SSM infection. This result, and increased FRC infection after SSM depletion (Fig 8), argued that FRC infection is normally suppressed by IFN-I from MCMV-exposed SSM.

## Discussion

LN survey extracellular fluid returning to the blood. SSM remove particulate antigens such as viruses. However viruses that replicate in SSM, or bypass them by infecting FRC, mandate additional defences. MCMV, like HCMV, has a broad tropism, encompassing fibroblasts, macrophages and endothelial cells, and lymph-borne MCMV directly infected both SSM and FRC. Thus, the need to contain this infection preceded adaptive immunity. Containment depended primarily on IFN-I, which restricted SSM and FRC infections despite viral IFN-I evasion [42]. Innate immunity is inherently polylithic, and IFN-I was not the sole SSM-based defence: NK cell recruitment was also important. Nor were SSM the sole IFN-I producers at the subcapsular sinus: pDC also contributed. But IFN-I and SSM were major players, and boosting their engagement provides a potential route to better infection control.

Most analyses of SSM anti-viral functions have infected mice with model, xenogenic pathogens such as VSV [18]. VSV replicates rapidly in many cell types, including SSM. Limited replication of the mouse-adapted, macrophage-tropic MCMV in SSM was consequently surprising. However filtering macrophages also restrict the macrophage-tropic murine pathogens lymphocytic choriomeningitis virus [43] and murine coronavirus [44] via IFN-I. As IFN-I contributes to myeloid cell homeostasis [45], incomplete evasion may be a viral compromise necessary to exploit myeloid cells for dissemination and persistence [4].

MCMV replicated preferentially in FRC. These provide LN structure, for example supporting the conduits that take low molecular weight proteins from the afferent lymph to near high endothelial venules for antigen presentation [11]. The conduits are too small to transport virions, but the lining FRC provide a route to the blood, and restricting acute FRC infection was an important part of SSM-mediated host defence. When the SSM, IFN-I or NK cell components of host defence were compromised, FRC seemed to provide the main additional site of viral replication.

The greater effects on MCMV of IFNAR blockade than pDC depletion, and of IFNAR blockade plus SSM depletion than SSM depletion alone, implied redundancy in IFN-I production between SSM and pDC. Medullary sinus and medullary cord macrophages [38] and conventional dendritic cells may also contribute, as may resident stromal cells and recruited inflammatory cells. Most pDC are migratory and recruited to inflammatory sites [29]. Thus their response can take time to develop. The early (day 1) inhibition of MCMV infection by IFN-I, including inhibition of viral reporter gene expression in the first cells encountered, was consistent with a key role for resident SSM. SSM depletion possibly under-estimated their contribution, as the ensuing inflammation could recruit other, compensatory IFN-I-producing cells. We envisage that SSM are constitutively in a high state of readiness to mount IFN-I responses to captured virions, whereas immigrant cells function more as a back-up should SSM prove insufficient.

NK cell recruitment was another important back-up to the SSM IFN-I response. Whole organ titers have established the importance of NK cells for MCMV control [15, 31, 36] and the impact of viral evasion [46]. What infected cell types NK cells target has been less clear. Although lymph-borne virions infected large numbers of SSM, the SSM contribution to new virion production was relatively low. FRC seemed to be the main source. A similar situation occurs in the lungs, where alveolar macrophages are prominent infection targets but most new virus seems to come from type II alveolar epithelial cells [47]. Consequently FRC were an important NK cell target. When IFNAR was blocked, increased FRC infection and NK cell recruitment led to tissue damage. This destructive capacity of NK cells could explain why inflammation around the subcapsular sinus compromises subsequent immune responses [48].

**Fig 12:**
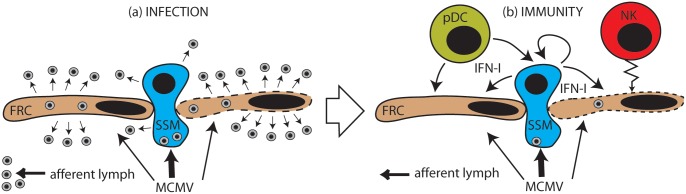
Schematic representation of MCMV escape from the LN subcapsular sinus. Most virions in the afferent lymph are captured by SSM, but some infect FRC. In the absence of IFN-I **(a)**, FRC infection is highly productive, SSM infection rather less so. Virions are released into the LN to infect new FRC and myeloid cells, and also back into the lymph flow, whence they can reach the blood. To counter this **(b)**, IFN-I from SSM capturing virions inhibits infection in both SSM and FRC. pDC also produce IFN-I. They may receive infected cell debris and cytokines via the FRC conduits. Inflammatory mediators also recruit NK cells to kill infected FRC. Thus MCMV propagation is largely suppressed.

Fig 12 summarizes what we now know of MCMV infection and control at the LN subcapsular sinus.

Most virions in the afferent lymph are captured by SSM, but some directly infect FRC. Without IFN-I and NK cells, FRC infection is highly productive. New virions then spread to the liver and spleen via the efferent lymph and blood. Their infections probably follow a similar pattern, with Kupffer cells and MZM taking the place of SSM [49, 50]. SSM restrict their own infection and that of FRC through IFN-I, and possibly also other pro-inflammatory cytokines such as IL-18 [37]; and they recruit cellular effectors, including NK cells which kill infected FRC.

Protection against VSV depends on blocking acute neuronal infection; protection against MCMV depends on reducing its spread to sites of persistence, such as the bone marrow and salivary glands. Thus a key question now is whether recruiting innate effectors more rapidly and in amplified form can reduce long-term systemic CMV loads. IFN-I alone is unlikely to protect against chronic infection. However a capacity to amplify and recruit innate responses at the LN subcapsular sinus, for example through CD4^+^ T cell-derived IFN-γ [49], may be an important property of vaccine-induced immunity.

## Materials and Methods

### Mice

BALB/c, C57BL/6J, LysM-cre [51], CD11c-cre [52] and IFNAR^flox/flox^ [53] mice were maintained at University of Queensland animal units and used when 6–12 weeks old. For myeloid cell-specific IFNAR disruption, LysM-cre mice were back-crossed onto IFNAR^flox/flox^. Virus was injected i.f. (10^6^ p.f.u. in 50μl) under isoflurane anesthesia. For luciferase imaging, mice were given 2mg luciferin i.p. and monitored for light emission by charge-coupled device camera scanning (Xenogen, IVIS-200). Phagocytic cells were depleted with 50μl i.f. clodronate-loaded liposomes (http://clodronateliposomes.org) [39] 1 and 3 days before infection. Depletion was confirmed in uninfected mice by a loss of CD169 expression around the PLN subcapsular sinus [6, 22]. NK cells were depleted from C57BL/6 mice with anti-NK1.1 mAb PK136 (200μg i.p., Bio-X-cell) and from BALB/c or C57BL/6 mice with anti-asialo-GM1 pAb (100μl i.p., Wako Chemicals). Each was given 1 and 3 days before infection and every 2 days thereafter. Cell depletion was >90% effective, as measured by immunostaining of tissue sections with anti-NKp46 antibody. IFN-I signalling was blocked with anti-IFNAR mAb MAR1-5A3 (200μg i.p., Bio-X-cell), given 1 day before infection and every 2 days thereafter. pDC were depleted with anti-Bst2 mAb BX444 (250μg i.p., Bio-X-Cell), 1 and 3 days before infection and every 2 days thereafter. Cell depletion was 85–90% effective, as measured by flow cytometry for Bst-2 and SiglecH. Experimental groups were compared statistically by two-tailed unpaired Student’s t test unless stated otherwise.

### Ethics statement

All experiments were approved by the University of Queensland Animal Ethics Committee (Licence numbers 218/15, 391/15, 479/15) in accordance with the *Australian Code for the Care and Use of Animals for Scientific Purposes* and regulated under the Queensland *Animal Care and Protection Act* (2001).

### Cells and viruses

MCMV strain K181 (MCK2^+^) was used throughout. All modified viruses were derived from MCMV K181 by homologous recombination. For live imaging we used a derivative (MCMV-LUC) that expresses firefly luciferase via autocatalytic release from a fusion protein with the lytic cycle M78 gene product [54]. For floxed reporter gene switching we used MCMV-GR, which has an HCMV IE1 promoter-driven cassette inserted into M157 [6]. The cassette encodes a floxed GFP gene upstream of a nuclear-targeted tdTomato gene. Thus, cre irreversibly switches MCMV-GR from green to nuclear red fluorescence. Switched and unswitched MCMV-GR show no difference in host colonization. MCMV with the essential virion glycoprotein L (gL) gene disrupted by insertion of an HCMV IE1 promoter-driven β-galactosidase expression cassette into M115 [6] was propagated on gL-expressing NIH-3T3 cells. All other viruses were propagated on NIH-3T3 cells (American Type Culture Collection CRL-1658). Infected cells were cleared of cell debris by low speed centrifugation (500 x *g*, 10min), then virus was concentrated by ultracentrifugation (35,000 x *g*, 2h). Infectious virus was plaque assayed on murine embryonic fibroblasts. Cells were grown in Dulbecco’s modified Eagle’s medium supplemented with 2mM glutamine, 100IU/ml penicillin, 100μg/ml streptomycin and 10% fetal calf serum.

### Immunostaining

Organs were fixed in 1% formaldehyde-10mM sodium periodate-75mM L-lysine (24h, 4°C), equilibrated in 30% sucrose (18h 4°C), then frozen in OCT. Sections (6μM) were air dried (1h, 23°C), washed 3x in PBS, blocked with 0.3% Triton X-100-5% normal goat serum (1h, 23°C), then incubated (18h, 4°C) with antibodies to B220 (rat mAb RA3-6B2, Santa Cruz Biotechnology), CD68 (rat mAb FA-11), ER-TR7 (rat mAb), CD11c (hamster mAb N418), SiglecH (rat mAb 440c), β-galactosidase (chicken pAb), Caspase 3, Lyve-1 (both rabbit pAb, AbCam), CD206 (rat mAb MR5D3), CD169 (rat mAb 3D6.112, Serotec); NKp46 (Rat Mab 29A1.4, Biolegend), MCMV IE1 pp89 (mouse mAb Croma101) [55] and MCMV virion antigens (rabbit pAb, raised in house by subcutaneous inoculation with MCMV K181 propagated in NIH 3T3 cells). After incubation with primary antibodies, sections were washed x3 in PBS, incubated (1h, 23°C) with combinations of Alexa 568- or Alexa 647-conjugated goat anti rat IgG pAb, Alexa 647-conjugated goat anti-mouse IgG1 pAb, (Life Technologies), and Alexa 488- goat anti-chicken pAb (Abcam), then washed x3 in PBS, stained with Hoechst 33342 and mounted in ProLong Gold (Life Technologies). Fluorescence was visualised with a Zeiss LSM510 confocal microscope and analysed with Zen imaging software.
